# Dr. John Deans Scouller

**Published:** 1917-06

**Authors:** 


**Dr. John Deans Scouller,** Buffalo 1870, Supt. of the State Reformatory at Pontiac, Ill., died Mch. 31, after an operation, aged 81.

